# Measuring Phonon Mean Free Path Distributions by Probing Quasiballistic Phonon Transport in Grating Nanostructures

**DOI:** 10.1038/srep17131

**Published:** 2015-11-27

**Authors:** Lingping Zeng, Kimberlee C. Collins, Yongjie Hu, Maria N. Luckyanova, Alexei A. Maznev, Samuel Huberman, Vazrik Chiloyan, Jiawei Zhou, Xiaopeng Huang, Keith A. Nelson, Gang Chen

**Affiliations:** 1 Department of Mechanical Engineering, Massachusetts Institute of Technology, Cambridge, Massachusetts 02139, United States; 2 Department of Chemistry, Massachusetts Institute of Technology, Cambridge, Massachusetts 02139, United States; 3 Department of Mechanical and Aerospace Engineering, University of California, Los Angeles, CA 91106, United States

## Abstract

Heat conduction in semiconductors and dielectrics depends upon their phonon mean free paths that describe the average travelling distance between two consecutive phonon scattering events. Nondiffusive phonon transport is being exploited to extract phonon mean free path distributions. Here, we describe an implementation of a nanoscale thermal conductivity spectroscopy technique that allows for the study of mean free path distributions in optically absorbing materials with relatively simple fabrication and a straightforward analysis scheme. We pattern 1D metallic grating of various line widths but fixed gap size on sample surfaces. The metal lines serve as both heaters and thermometers in time-domain thermoreflectance measurements and simultaneously act as wire-grid polarizers that protect the underlying substrate from direct optical excitation and heating. We demonstrate the viability of this technique by studying length-dependent thermal conductivities of silicon at various temperatures. The thermal conductivities measured with different metal line widths are analyzed using suppression functions calculated from the Boltzmann transport equation to extract the phonon mean free path distributions with no calibration required. This table-top ultrafast thermal transport spectroscopy technique enables the study of mean free path spectra in a wide range of technologically important materials.

Thermal transport in semiconductors and dielectrics generally involves the accumulative contributions of phonons spanning a wide range of mean free paths (MFPs)^1^,^2^,^3^,^4^,^5^,^6^,^7^. The thermal conductivity accumulation function, 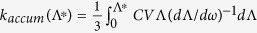, where *C*, *V*, and Λ are the phonon mode dependent specific heat, group velocity and MFP, respectively, is the key metric to describe the contributions of phonons with different MFPs to heat transport under the relaxation time approximation^8^. *k*_*accum*_(Λ^*^), yielding the contributions of phonons with MFPs less than a threshold value Λ^*^, essentially describes the distribution of phonon MFPs contributing to a material’s thermal conductivity. Knowing materials’ MFP distributions is critically important for both fundamental description of microscopic energy flow in materials^9^,^10^,^11^ and many practical applications, including optimizing thermal management in microelectronics^12^,^13^,^14^,^15^ and tailoring thermal conductivity in nanostructured thermoelectric materials^16^,^17^,^18^,^19^,^20^,^21^,^22^. At present, the thermal conductivity accumulation function can be computed from first-principles density functional theory (DFT) based calculations without assuming any fitting parameter^23^,^24^,^25^. However, accurate DFT calculation is currently limited to simple single crystalline materials. Computing MFP distributions in complex materials, such as nanocomposites and nanostructured thermoelectric materials, is still computationally prohibitive.

In principle, the MFP distribution can be obtained from experimental measurements of phonon lifetimes and dispersion by inelastic neutron scattering^26^,^27^. However, this requires measurements of phonon lifetimes across the entire Brillouin zone. Due to instrumental limitations of inelastic neutron scattering, this has been attempted so far only in a few materials with short phonon lifetimes^26^,^27^. Moreover, inelastic neutron scattering is limited to characterizing single crystals and requires large-scale central facilities. An emerging optical desk-top approach focuses on utilizing quasiballistic phonon transport, created when characteristic length scales become comparable to the phonon MFPs, to map out the MFP distributions^2^,^3^,^4^,^5^,^6^. The idea is to measure transport across a characteristic length *w*, such that the contributions of phonons with MFPs larger than *w* to the thermal transport are suppressed. By varying *w*, information on relative contributions of phonons with different MFPs can be inferred. The characteristic length can be the size of the heat source^2^,^3^,^6^,^15^, the experimentally determined thermal transport distance^4^, the thermal diffusion lengths traversed during an experimentally determined time period^1^,^5^,^28^, or the dimension of a nanostructure^7^. Typically, this approach relies on the deviation of the thermal transport from the Fourier law at small length scales^29^. In diffusive transport where the heat source dimension is much larger than the phonon MFPs, as shown in Fig. 1(a) for the experimental configuration used in this work, phonons undergo sufficient scattering to maintain local thermodynamic equilibrium and the heat conduction is accurately described by Fourier diffusion theory. In quasiballistic transport where the heat source dimension is comparable with some phonon MFPs (Fig. 1(b)), long-MFP phonons do not experience scattering as inherently assumed by Fourier’s law and no local thermal equilibrium can be established, leading to the breakdown of the heat diffusion theory^29^. Consequently, the measured thermal conductivity incorporates a quasiballistic thermal resistance that depends upon the characteristic thermal transport length in the measurement^2^,^3^,^6^,^29^. By systematically varying the characteristic length to sample phonons of different MFPs and using a suppression function to connect the length-dependent thermal conductivity to MFPs, the phonon MFP distributions in the target materials can be extracted^30^,^31^.

One nontrivial challenge to probe phonon MFPs is to reduce the characteristic length scale of the measurement such that quasiballistic transport can be sampled. In a laser-based experiment such as time-domain thermoreflectance (TDTR)^32^,^33^, the heater size can be readily varied by changing the heating laser spot size^3^, but the smallest achievable heater size is diffraction-limited by the optical wavelength. In order to probe phonon MFPs in the nanometer range, one can fabricate nanometer-sized heaters by depositing metal dots or lines on the sample surface^2^,^6^,^15^. For TDTR measurements, the use of nanodots or nanolines works well with transparent materials such as sapphire^6^. However, for opaque materials, the excitation laser light will be absorbed not only by the metal heaters but also by the material itself, complicating the analysis. Additionally, if the material is a semiconductor, photo-excited carriers will contribute to the TDTR signal, complicating the analysis further. Hu *et al.* used a bilayer hybrid nanostructure to protect the substrate from direct heating^6^; however, the hybrid approach can hardly be recommended as a generic MFP spectroscopy tool due to the fabrication complexity. Measuring diffraction of an extreme UV probe^2^,^15^, predominantly sensitive to the photothermally induced surface displacement, largely alleviates the problems associated with electronic excitation and moderate substrate heating.

Another nontrivial challenge is the mapping of experimentally measured effective thermal conductivities to the phonon MFP distribution in the material under study. In early studies, the analysis was done using simple models based mainly on physical intuition, such as cutting off the contributions of phonons with MFPs exceeding the characteristic length^1^,^3^. The need for a more quantitative approach based on the Boltzmann transport equation (BTE) has been well recognized^6^,^7^,^31^, but such an approach has not yet been consistently implemented for extracting the MFP distribution from nondiffusive thermal transport measurements. One exception to the above statement is ref. 7 where thermal transport in thin membranes was measured in the diffusive regime and the well-established Fuchs-Sondheimer model was used to reconstruct the MFP distribution. However, the approach of ref. 7 requires the fabrication of membranes spanning a broad range of thicknesses commensurate with the phonon MFPs, making it impractical as a generic MFP spectroscopy tool.

In this report, we describe a nanoscale MFP spectroscopy technique suitable for many materials, transparent or opaque, that overcomes the above-mentioned challenges. The key feature of our approach is that the 1D array of metal lines used as nanoscale heaters is designed with subwavelength gaps between the lines in order to simultaneously function as a wire-grid polarizer, insulating the substrate from both excitation and probe light, so that the standard TDTR measurement approach can be used without complications due to direct substrate heating and electron-hole generation. Compared with the EUV diffraction method^2^,^15^, our present approach permits measurement of thermal transport in similar nanostructured samples using more readily available optical probe wavelengths; the requirement for a subwavelength gap dimension restricts our sensitivity to submicron transport lengths that are crucially important and generally inaccessible optically. Furthermore, we develop a consistent BTE-based approach for reconstructing the MFP distribution from the experimental data based on a generalization of the suppression function method proposed in ref. 31. We demonstrate the reconstruction of phonon MFP distributions in crystalline silicon at different temperatures without any fitting parameters or calibration procedures. The resulting MFP spectra agree quite well with first-principles calculations. The experimental simplicity combined with the straightforward analysis algorithm makes our approach applicable to a wide range of materials at the nanoscale.

## Results

### Sample design and experimental setup: insulating the substrate from optical excitation and probing

**Figure 1:**
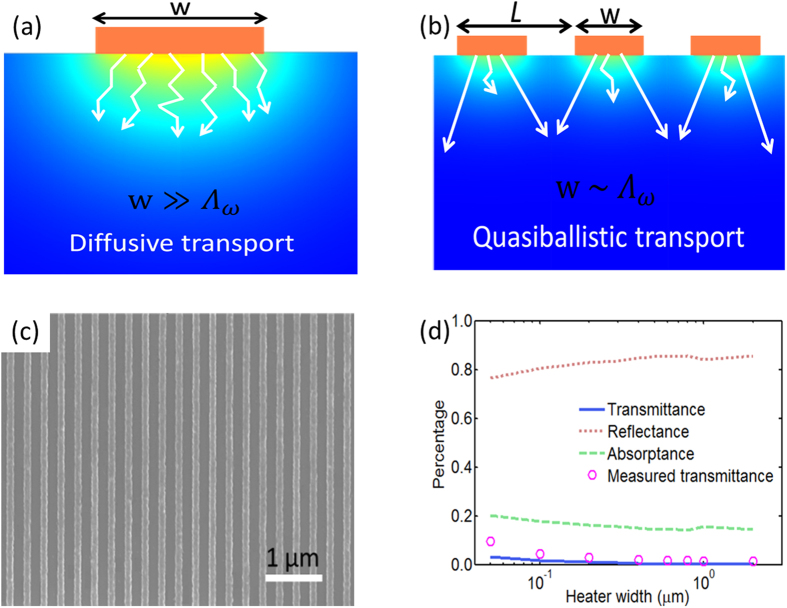
(**a**) Heat transport is diffusive when heater size *w* is much larger than phonon MFPs. (**b**) Heat transport becomes quasiballistic when heater line width *w* is comparable to phonon MFPs. (**c**) SEM image of a typical aluminum grating on silicon substrate. (**d**) Simulated and measured transmittance of Al grating on sapphire versus the grating line width.

Our samples, as shown in Fig. 1(b), consist of an array of closely spaced metal lines of various widths on top of the substrate under study. This experimental geometry is much simpler than the 2D hybrid nanostructure developed previously in our group^6^. To avoid direct substrate heating due to laser transmission through the openings between neighboring metal lines, we keep the spacing between neighboring lines to be constant at approximately 150 nm, much smaller than the laser wavelength (~790 nm), while systematically varying the heater size (defined by the metal line width). This sample structure differs from previously used structures in that prior experimental studies typically kept the filling fraction (defined as the ratio of the grating line width to the grating period) constant^2^,^6^. Our grating line width varies from 10 μm down to 50 nm, implying a changing filling fraction across gratings with different line widths. The line width and spacing for all the grating patterns were measured using scanning electron microscopy (SEM). The thickness of the metal layers was measured using atomic force microscopy (AFM). Figure 1(c) shows a typical SEM image of a fabricated aluminum grating on a crystalline silicon substrate. The metallic gratings act as optical wire-grid polarizers which effectively prevent laser light with polarization parallel to the grating lines from passing through the openings between neighboring lines.

To examine the laser energy transmission to the substrate surface, we performed optical simulations using COMSOL Multiphysics software package^34^ to obtain the laser transmittance through a metal grating. The simulation domain consists of an aluminum grating on a sapphire substrate, mimicking the experimental sample configuration. The optical simulation results, as shown in Fig. 1(d), suggest that a spacing of 150 nm is sufficient to achieve negligible direct laser transmission to the substrate. This is expected since the pump and probe wavelengths (~785 nm) are much longer than the spacing between neighboring grating lines and the extraordinary transmission phenomenon is not strong in Al^35^. The measured transmittance of Al gratings on sapphire as a function of line width for approximately 150 nm spacing between neighboring grating lines is also shown in Fig. 1(d) for comparison. The measured transmittance data, although being approximately 3 times that of simulation data for the smallest line width structure due to fabrication irregularities, confirm insignificant laser transmission to the substrate.

We used a two-tint TDTR setup^32^,^33^,^36^ with 791 nm pump and 780 nm probe wavelengths for the thermal conductivity measurements. Both the pump and probe beams are linearly polarized with their **E** fields parallel to the metallic grating. This ensures that our measured thermoreflectance signal comes only from the metal grating due to the insignificant light transmission to the substrate. The substrate effective thermal conductivity and the interfacial thermal conductance between the metal transducer and the substrate are extracted by fitting the experimental reflectance signal with the model prediction based on the heat diffusion theory^32^.

### Length-dependent thermal conductivity measurement results

**Figure 2:**
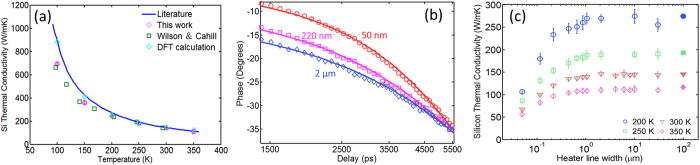
(**a**) Measured silicon thermal conductivities (circles and squares), DFT computed thermal conductivity (diamonds), and the literature data (solid line, ref. 38). (**b**) Representative traces of measured room temperature TDTR reflectance signals (circles) and best model fits (solid lines) for three heater widths: 50 nm, 220 nm, and 2 μm. The effective thermal conductivities for these three samples are approximately 66.0 W/mK, 120.0 W/mK, and 140.0 W/mK, respectively. (**c**) Silicon effective thermal conductivities versus heater width at 200 K, 250 K, 300 K and 350 K, respectively. The error bars represent standard deviations in the measured thermal conductivities. The filled dots represent silicon bulk thermal conductivity from literature (ref. 38) at four different temperatures (circle: 200 K; square: 250 K; triangle: 300 K; diamond: 350 K).

We first measured the effective thermal conductivity of silicon at various temperatures using a 30 μm pump spot size on a continuous aluminum film. Figure 2(a) shows the comparison of temperature-dependent silicon thermal conductivities from TDTR measurements^37^, DFT calculations and literature^38^,^39^. We refined our DFT calculation reported before^24^ by using a much finer k mesh in the reciprocal space and the computed thermal conductivities are consistent with the literature data for the entire examined temperature range. Above 200 K, our measurement results agree well with literature and prior TDTR measurements^37^ (ref. 37 used 25 μm pump diameter). However, below 200 K, our measured thermal conductivities fall below the literature value due to quasiballistic transport induced by the finite pump spot size^40^ and again agree well with prior measurement data^37^.

We then measured the heater-width-dependent thermal conductivities of silicon samples with aluminum gratings at different temperatures. Since the heating laser spot diameter is typically orders of magnitude larger than the grating line width, the diffusion heat transfer model used for the grating samples assumes two-dimensional thermal transport in the plane perpendicular to the metal grating and accounts for both the heater line width and the spacing between neighboring heaters. Representative traces of the measured phase signals at room temperature and the corresponding model fits for 50 nm, 220 nm and 2 μm heater widths are shown in Fig. 2(b). The fitting quality is excellent for all the heater line widths, indicating that our heat transfer model incorporating an effective substrate thermal conductivity and interface conductance describes well the thermal transport occurring in the TDTR experiments. The effective thermal conductivity decreases dramatically with decreasing heater width. At a very large heater width (2 μm), the measurement returns an effective silicon thermal conductivity very close to the bulk value, indicating diffusive thermal transport in the substrate. At a 220 nm heater width, the effective thermal conductivity is approximately 82% of the bulk value, which suggests that the transport becomes quasiballistic and the experiment measures an additional ballistic resistance^2^,^3^,^6^. The quasiballistic effect becomes stronger with decreasing heater width, as verified by the constantly decreasing effective thermal conductivity. At a 50 nm heater line width, the thermal conductivity drops to ~46% of the bulk value. We should caution that the observation of near-diffusive transport when *w* = 2 μm does not suggest the maximum phonon MFP in silicon is less than 2 μm since the onset of the quasiballistic transport depends upon the heater line width, the filling fraction of the heater array and the phonon MFP distribution^41^.

The effective thermal conductivities of silicon versus heater line width at four different temperatures (circle: 200 K; square: 250 K; triangle: 300 K; diamond: 350 K) are shown in Fig. 2(c). At room temperature, the transition from diffusive transport to quasiballistic transport occurs around 1 μm, below which length scale quasiballistic effect becomes increasingly stronger with decreasing heater width. Again, the observed diffusive transport above 1 μm originates from the weak sensitivity of the current measurement approach to probe MFPs larger than several microns and does not indicate that the intrinsic phonon MFP in the sample is less than 1 μm, as will be discussed later. In fact, earlier measurements at room temperature^4^,^6^ and reduced temperatures^3^ have shown that silicon exhibits non-diffusive transport at length scales significantly greater than 1 μm. To a first approximation, phonons in the silicon substrate can be divided into two groups: a diffusive group with MFPs shorter than the heater width and a ballistic group with MFPs longer than the heater width^42^. The observed increasing ballistic resistance originates from an increasingly larger portion of phonons joining the ballistic group as the heater width is reduced. Varying the heater line width in a systematic manner helps sample different MFP phonons’ contribution to thermal transport, making it possible to extract the phonon MFP distribution information from the size-dependent thermal conductivities.

### MFP spectra reconstruction and comparison with DFT calculations

To gain insight into which phonon MFPs are responsible for heat conduction in the substrate, we follow the approach proposed by Minnich^31^. To extract the intrinsic phonon MFP distribution from the measured length-dependent thermal conductivities *k*_*eff*_, Minnich^31^ introduced a heat flux suppression function to relate the measurement results to the MFPs: 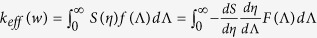, where 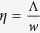 is the ratio of spectral MFP to the characteristic thermal length *w*, *S*(*η*) is the suppression function, 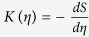 is the kernel function, *f*(Λ) is the differential phonon MFP distribution, and *F*(Λ) is the cumulative phonon MFP distribution (i.e. *k*_*accum*_(Λ)). *F*(Λ) is related to *f*(Λ) through: 
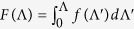
. The suppression function represents the reduction in phonon heat flux from different MFP phonon modes for a given material system with respect to Fourier’s law prediction. The key assumption is that the suppression function depends only on the experimental configuration (i.e. the sample structure and the distribution of the heat sources), while the dependence of the measurement on the material properties is entirely described by the MFP distribution. This assumption enables extracting the MFP distribution from the measurement results without any prior knowledge of the phonon properties of the sample material. Moreover, in this case the same suppression function applies to a model “gray-body” medium in which all phonons have the same MFP. Thus the suppression function can be calculated from the gray-body BTE^43^. In reality, the above assumption is not entirely accurate^43^; however, it has been shown to work well, in comparison to the accurate spectral BTE solution, in describing thermal transport induced by a spatially periodic heat source^43^. We will use this assumption here while realizing that it is not rigorous.

Minnich’s formulation of the MFP reconstruction algorithm^31^ also assumes that the measurement is characterized by a single length parameter *w* such that the suppression function is a function of the single variable 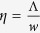. This is rarely the case in a real experiment: in fact, the transient grating measurements of thin membranes considered in ref. 31 involved two characteristic lengths, i.e. the membrane thickness and the transient grating period. Likewise, our experiments involve two characteristic geometric lengths: the heater line width *w* and the array period *L*. However, the approach developed by Minnich^31^ can be used in a more general way not requiring that the suppression function for all measurements be given by a single function of a single nondimensional variable. Consider N measurements on the same or different samples, with an effective thermal conductivity *k*_*i*_ in the *i*-*th* measurement described by the suppression function *S*_*i*_(*η*): 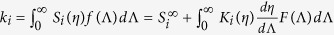, where *K*_*i*_(*η*) is the kernel function for the *i*-th measurement. The second equality is obtained through integration by parts and note that *F*(0) = 0 and *F*(∞) = 1. Thus we generalize Minnich’s equation presented earlier by allowing the suppression function to be different for each measurement (the possibility of such generalization has been in fact pointed out by Minnich^31^). A further generalization is made by allowing the suppression function to have nonzero residual suppression at the limit of large 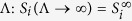. Although the residual suppression factor is zero for some experimental configurations, it can be finite and significant for other geometries, such as the ones used in this work as discussed below.

The suppression functions for our sample geometries depend on two non dimensional variables: the ratio of MFP to heater width 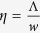 and filling fraction 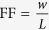, where *L* is the heater array period. The filling fraction takes into account the impact of the heater array periodicity on the heat transfer regime in the underlying substrate. The relevant suppression function for the measurement on sample *i* with filling fraction FF_*i*_ is *S*_*i*_ = *S*(*η*, FF_*i*_). The suppression functions are calculated from the gray-body phonon BTE and are subsequently combined with the measurement results to determine the MFP distributions. The details of solving the phonon BTE for our experimental geometry to obtain the suppression functions are presented elsewhere^41^. Briefly, an initial temperature pulse is applied to the line heater array to drive the heat flow between the heater and the substrate. Periodic boundary condition is implemented to take into account the periodicity of the heating nanostructure. For each heater width, the surface temperature of the heater is computed by solving the phonon BTE and matched to the solution from the heat equation to obtain the length-dependent substrate effective thermal conductivity. We find that the surface specularity (defined as the fraction of specularly reflected phonons at a given boundary) has a negligible effect on the simulation results.

**Figure 3:**
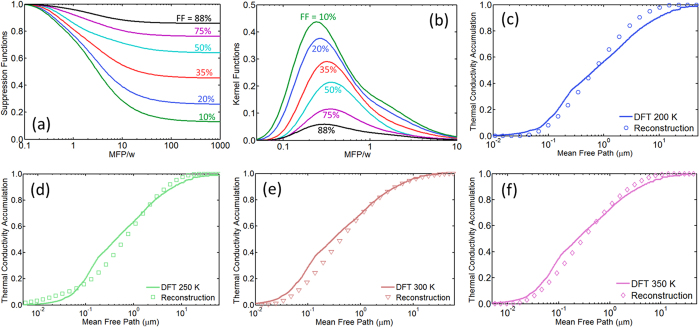
(**a**) Computed heat flux suppression functions at different filling fractions based on solving the phonon Boltzmann transport equation. (**b**) Computed kernel functions versus filling fractions. (**c**–**f**) Comparison of experimentally reconstructed silicon MFP distributions and predictions from DFT calculations at four different temperatures. The MFP distribution describes the fractional thermal conductivity contribution from thermal phonons with MFPs shorter than a prescribed value.

The computed suppression functions across a range of filling fractions, as shown in Fig. 3(a), capture the transition from totally diffusive transport regime to strongly ballistic transport regime. In the diffusive limit, all the phonons contribute to thermal conductivity as described by the heat diffusion theory and the suppression factor is unity regardless of the filling fraction, whereas in the quasiballistic limit, long-MFP phonons’ contributions to heat transport are suppressed and the suppression factor depends on both the ratio of MFP to heater width 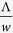 and the filling fraction FF. Depending on the spacing between neighboring heaters, thermal transport in the substrate external to the heater array may differ significantly from highly ballistic heat spreading from an isolated nanoscale hot spot^15^,^41^. Given the heater size, the thermal transport near an individual heater is weakly affected by the presence of its neighboring heaters in the case of small filling fraction, whereas for a large filling fraction, the thermal transport near one heater is strongly influenced by the presence of neighboring heaters. The BTE simulation returns the bulk substrate thermal conductivity for a continuous film heater (FF = 100%) that can be viewed as a superposition of an infinite number of isolated point heat sources on the substrate, suggesting that superposition of closely spaced ballistic heat sources recovers the familiar diffusive transport in the underlying substrate. As the filling fraction approaches 100%, the suppression function approaches unity for all phonon MFPs. Figure 2(c) shows that our measurements yield the bulk thermal conductivity values for *w* > 1 μm, indicating that for filling fractions above 90% the measurements are no longer sensitive to non-diffusive transport. For the same ratio of phonon MFP to heater size, the BTE simulation shows that a smaller filling fraction leads to stronger ballistic transport as demonstrated by Fig. 3(a)^41^. The residual suppression function value that occurs when the phonon MFP is much larger than the heater width increases toward unity with increasing filling fraction. Figure 3(a) clearly shows that phonons with MFPs longer than the heater line width still carry significant amount of heat. In fact, our results show that significant residual suppression exists for finite filling fractions even when 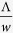 approaches infinity.

After suppression functions for all the samples are calculated, a convex optimization scheme is used to reconstruct the phonon MFP distribution^31^,^43^. For our sample geometry, the kernel functions, as shown in Fig. 3(b), span just under two orders of magnitude from approximately 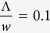 to 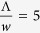, meaning that the thermal conductivity measurement for a certain heater width *w* contains MFP distribution information ranging from approximately one tenth of the heater width to five times that of the heater width. Since our minimum heater line width is 50 nm, our MFP reconstruction scheme is sensitive to the cumulative MFP distribution much smaller than 50 nm (down to ~5 nm). Figure 3(c–f) compare the reconstructed silicon MFP distributions at four different temperatures with DFT calculations using a very dense k mesh (64 × 64 × 64 k meshes) in the reciprocal space. The reconstructed phonon MFP distributions agree well with DFT predictions for all the studied temperatures. The agreement demonstrates the viability of the current thermal conductivity spectroscopy technique. We note that the non-uniformity in the grating spacing generally does not affect the reconstructed MFP distributions significantly since it is much smaller than the grating spacing and line width. In addition, the crossover between the DFT calculated MFP distributions and the reconstructed MFP distributions, if any, does not have significant physical implication except that it means that both DFT and experimental reconstruction predict the same fractional thermal conductivity contribution from phonons with MFPs shorter than the value at the crossover.

## Discussion

The described thermal conductivity spectroscopy technique is a general method for studying phonon MFPs in a wide range of materials. The ability to obtain MFP spectra using this approach will lead to a better understanding of microscopic phonon-mediated heat transport in nanostructures and bulk materials and have important implications in many technological applications. Compared to our previous work using hybrid heater nanostructures^6^, the current approach utilizing 1D metal gratings greatly simplifies the fabrication processes. Given its sensitivity, our future work will focus on materials of practical interest, such as thermoelectrics. Future design of more efficient thermoelectric devices calls for a more adequate understanding of microscopic thermal transport in thermoelectric materials. As predicted by previous DFT calculations, most thermoelectric materials have dominant phonon MFPs in the tens to hundreds of nanometers range^44^. Therefore, our approach is well suited for measuring phonon MFPs in those materials for which *ab*-initio calculations are difficult and inaccurate due to the complex material structure. The ability to measure MFP distributions in thermoelectric materials may serve as a blueprint for engineers to tailor thermal conductivity for better device performance^16^,^17^,^44^. In addition, the reconstructed MFP distributions will aid device and structure design in optimizing thermal manangement of nanoelectronics^14^.

In summary, we developed a new implementation of a thermal conductivity spectroscopy technique which can be employed to probe phonon mean free paths in opaque materials down to tens of nanometers. The technique utilizes 1D metallic gratings with sub-optical-wavelength gaps between the metal lines to localize the heating to the metal lines and insulate the substrate from linearly polarized laser illumination. We demonstrated the technique by measuring the length-dependent effective thermal conductivities of crystalline silicon at various temperatures using the two-tint time-domain thermoreflectance method. We also generalized the MFP reconstruction scheme to measurements involving multiple characteristic lengths by recognizing the importance of residual suppressions in the limit of infinite phonon MFP, and we developed a consistent BTE-based procedure to extract the MFP distribution from the measured thermal conductivity data. The reconstructed phonon MFPs in silicon agree well with results predicted from first-principles calculations, without any fitting parameters. The agreement between the reconstructed MFP distributions and the DFT-based calculation results indicates that now we have both experimental and computational tools for studying nanoscale phonon MFPs in a wide range of materials of interest.

## Methods

### Sample fabrication and calibration

The samples consist of 1D aluminum gratings of various line widths sitting on top of crystalline silicon substrate. A standard electron beam lithography and liftoff method is used to pattern the gratings onto the substrate. To begin, we use BOE (Buffered Oxide Etch) to etch away the native oxide layer on the silicon substrate. Immediately following the oxide etch, we spin coat a thin layer of PMMA (Polymethyl methacrylate) resist (~260 nm) onto the substrate. Immediately after resist coating, the sample undergoes prebaking on a hot plate at 180 C for approximately 3 minutes. Then the resist is exposed by electron beam at 125 keV using an exposure machine ELIONIX in the Microsystems Technology Laboratory at MIT. After exposure, the resist is subsequently developed using 3:1 IPA:MIBK solution for 90 seconds, followed by an IPA (isopropyl alcohol) rinse and nitrogen blow-dry. We then use ebeam evaporator to deposit a thin aluminum film onto the silicon wafer. Following metal deposition, we use an acetone bath at room temperature to strip the remaining resist off the silicon substrate. Finally, the sample is rinsed by IPA and blown dry with nitrogen. The fabricated grating structures are calibrated using atomic force microscopy and scanning electron microscopy to obtain the pattern thickness and line width, respectively.

### Two-tint Time-domain thermoreflectance (TDTR) measurement

TDTR is a non-contact and non-invasive thermal measurement technique, particularly suitable for characterizing thermal properties of thin films, superlattices and bulk materials. In our two-tint TDTR measurements, sharp-edge optical filters are used to create spectrally distinct pump and probe beams. During the measurements, a modulated pump pulse train at ~791 nm impinges on the sample surface and is partially reflected and partially absorbed^32^. A second time-delayed probe pulse train at ~780 nm detects the change in the metal surface reflectance induced by the periodic pump heating. The delay time is regulated by varying the optical path length of the probe beam via a mechanical delay line. Both the pump and probe beams are linearly polarized parallel to the metal gratings to minimize direct substrate heating. The change in reflectance of the sample is measured as a function of the delay time between the pump and probe beams. Since the system is in the linear response regime, a change in the sample surface reflectance is linearly related to a change in the sample surface temperature, indicating that measuring reflectance change is equivalent to measuring change in surface temperature. A lock-in amplifier is used to detect the reflectance signal at the pump modulation frequency. The substrate effective thermal conductivity and the interfacial thermal conductance between the heater and the substrate are extracted by matching the experimental reflectance signal with the solution from the heat diffusion theory.

### Diffusion heat transfer model

In TDTR measurements, both the pump and probe spot diameters are much larger than the grating line width and period. As a result, we assume infinitely large pump and probe spot sizes in the diffusion heat transfer model. This approximation makes the thermal transport become two dimensional, neglecting the transport along the metal grating line direction. To mimic the one dimensional rectangular pump heating profile, we further assume that the in-plane thermal conductivity of the metal transducer is zero, thus heat can only diffuse in the cross-plane direction inside the metal layer. When we apply a heating profile that has the shape of the metal grating, no direct cross-talk between neighboring heaters exists. Heat transfer across the metal-substrate interface is described by an interface conductance G through *q* = G · ΔT, where *q* and ΔT are the interfacial heat flux and temperature difference, respectively. Under these approximations, the frequency domain thermal response can be obtained analytically by solving the heat equation using the transfer matrix method. Details on how to obtain the analytical frequency response solution can be found in the Supplementary Information. The effective substrate thermal conductivity and interface conductance are extracted by matching the measured reflectance signal with the prediction from the diffusion heat transfer model using a non-linear least square method.

### Mean free path reconstruction

The measured length-dependent effective thermal conductivities *k*_*eff*_(w, FF), the suppression functions *S*(*η*, FF) and the unknown cumulative MFP distribution *F*(Λ) are related through the following formula: 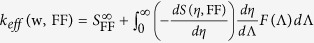, where *η* = Λ/w is the ratio of the phonon MFP to heater width, FF is the filling fraction and 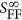 is the residual suppression factor for a particular FF. Since the experimental structures used have variable finite filling fractions, consistent suppression functions for different sample structures are used to perform MFP reconstruction. Linear interpolation is used to obtain the suppression functions at filling fractions not shown in Fig. 3(a). The desired cumulative MFP distribution *F*(Λ) can be extracted by solving an inverse problem with the measured effective thermal conductivities and the calculated suppression functions from Boltzmann transport equation. In practice, we use a convex optimization technique proposed by Minnich (ref. 31) to solve for *F*(Λ). Gaussian quadrature is used to discretize the MFP spectrum and evaluate the integral involving the cumulative distribution *F*(Λ). Since *F*(Λ) is a cumulative distribution function, we constrain it to increase monotonically from zero to one when the MFP varies from the minimum value to the maximum value. The convex optimization aims to minimize a penalty function 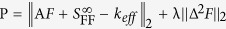, where 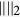 is the 2^nd^ norm, A is a 2D matrix as described in ref. 31, *F* is a vector at the discretized quadrature points, 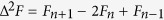, and λ is a smoothing factor which ensures the smoothness of the reconstructed MFP distribution. The smoothing factor weighs the accuracy penalty with the smoothness penalty. A too small smoothing factor will result in abrupt jumps in *F*(Λ) while a too big smoothing factor will lead to the optimization not solving the linear equations 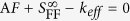. In principle, the smoothing factor should be set to the smallest value which still maintains the smoothness of the cumulative distribution function so that the optimization solves 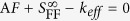 accurately. We use a smoothing factor of 0.15 to obtain the reconstructed cumulative MFP distribution.

## Additional Information

**How to cite this article**: Zeng, L. *et al.* Measuring Phonon Mean Free Path Distributions by Probing Quasiballistic Phonon Transport in Grating Nanostructures. *Sci. Rep.* **5**, 17131; doi: 10.1038/srep17131 (2015).

## Supplementary Material

Supplementary Information
