# Supplementary material for: Measuring Phonon Mean Free Path Distributions by Probing Quasiballistic Phonon Transport in Grating Nanostructures
Source: Sci Rep. 2015 Nov 27;5:17131. doi: 10.1038/srep17131 (PMC4661481; doi:10.1038/srep17131)
Supplement: Supplementary Information [file srep17131-s1.pdf]

Supplementary Information

Measuring Phonon Mean Free Path Distributions by Probing Quasiballistic Phonon Transport in Grating Nanostructures

L. Zeng[1*], K. C. Collins[1*], Y. Hu[3], M. N. Luckyanova[1], A. A. Maznev[2], S. Huberman[1], V. Chiloyan[1], J. Zhou[1], X. Huang[1], K. A. Nelson[2] and G. Chen[1,a)]

[1]Department of Mechanical Engineering, Massachusetts Institute of Technology, Cambridge, Massachusetts 02139, United States

[2]Department of Chemistry, Massachusetts Institute of Technology, Cambridge, Massachusetts 02139, United States

[3]Department of Mechanical and Aerospace Engineering, University of California, Los Angeles, CA 91106, United States

1. Sample structural design and calibration

Our samples consist of 1D aluminum metal grating of various line widths on top of crystalline silicon substrate, as sketched in Fig. S1a. The grating line width varies from tens of nanometers to tens of micrometers. For all the gratings of different widths, the gap (or spacing) between neighboring line heaters is fixed to be approximately 150 nm in order to minimize the direct substrate heating effect. Both the Boltzmann transport model and the diffusion heat transfer model take into account the metal line width and gap. We used scanning electron microscopy (SEM) and atomic force microscopy (AFM) to calibrate the metal line width and thickness of the fabricated heater nanostructures, respectively. One representative SEM image and one representative AFM image of the fabricated gratings are shown in Fig. S1b and Fig. S1c, respectively.

[a)] To whom correspondence should be addressed. Electronic mail: gchen2@mit.edu

* These authors contributed equally to this work.

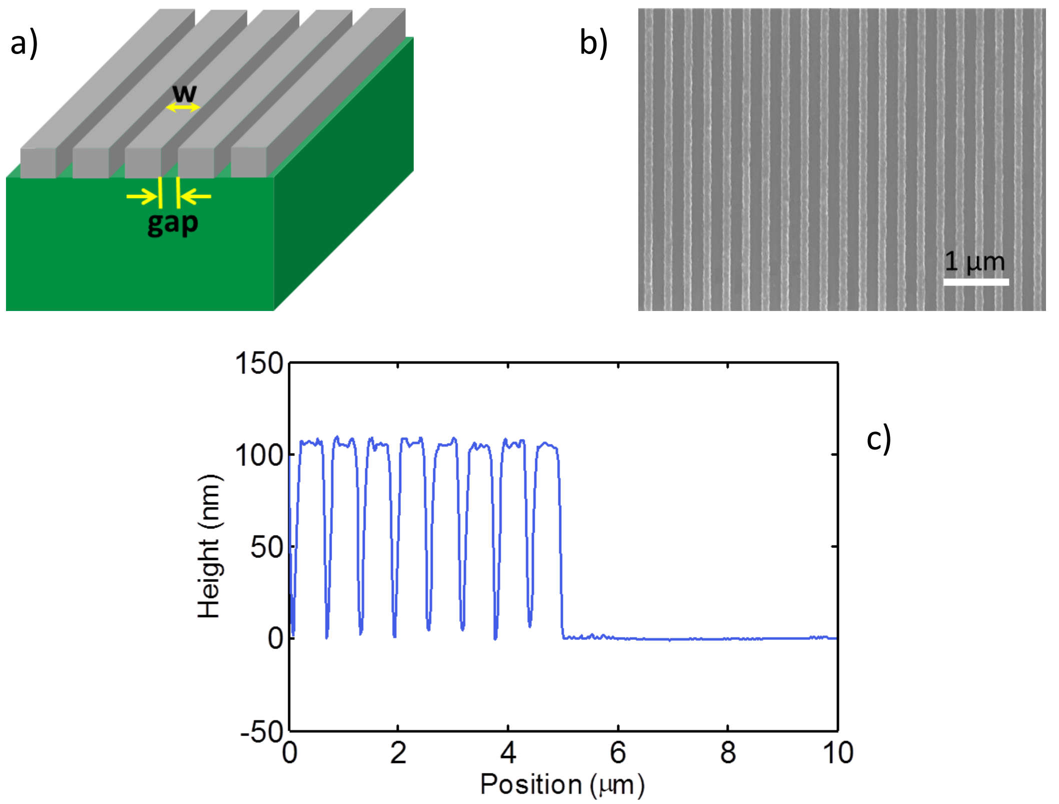

Figure S1. (a) Schematic illustration of sample structure. (b) SEM image of a typical aluminum grating on top of silicon substrate (scale bar = 1 µm). (c) AFM measurement of the metal thickness for a 450 nm wide grating on silicon substrate.

2. Grating transmittance simulation and measurement setup

To verify that the laser transmission through the opening between neighboring metal grating lines into the substrate is insignificant, we used COMSOL wave optics module to calculate the structure dependent transmittance[1]. The simulation domain, as sketched in Fig. S2a, consists of one unit cell of the grating due to the periodic nature of the grating structure. Floquet boundary conditions describing the periodicity are used for the periodic boundaries of the simulation domain[1]. We chose sapphire as the substrate underneath the Al metal grating since sapphire is transparent in our laser spectrum and allows us to directly compute the transmittance through the metal grating. The material optical properties used in the simulation are listed in Table S1. The wavelength is specified to be the center wavelength of our laser spectrum, 785 nm. In order to minimize laser transmittance, the polarization of the $\boldsymbol{E}$ field is aligned with the metal grating lines to mimic experimental conditions. The angle of incidence is zero (normal incidence). The metal thickness and the gap between neighboring metal lines are set to be 105 nm and 150 nm, consistent with our fabricated grating structures. As suggested by the optical simulation results shown in Fig. 1(d) in the manuscript, the transmittance through the grating to the substrate is indeed negligible.

In addition to optical simulations, we also set up a transmission platform and measured the laser transmittance through the grating structure for different grating line widths. The transmittance setup is sketched in Fig. S2(b). The laser first passes through a combination of a quarter wave plate which circularly polarizes the incoming light and a linear polarizer that linearizes the laser polarization in a selected direction. Then the laser beam is focused onto the sample with the patterned metal grating on top. The transmitted laser light is further focused into a photodetector which reads its intensity. The transmittance is defined as the ratio of the transmitted intensity through a metallic grating structure to the transmitted intensity through bare sapphire substrate. The measured transmittance as a function of grating line width is shown in Fig. 1(d) in the manuscript. The measured

result shows insignificant direct laser energy transmission, suggesting that the designed gratings indeed effectively minimize the laser transmittance to the substrate[2,3].

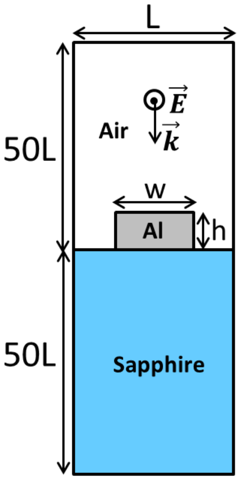

(a)

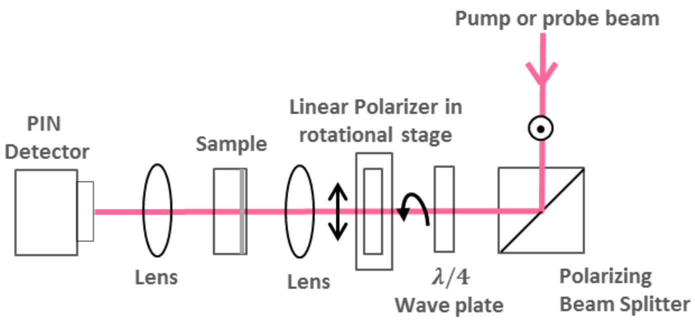

(b)

Figure S2. (a) Schematic illustration of optical simulation domain. (b) Schematic illustration of transmittance measurement setup.

Table S1. Material optical properties at 785 nm (source: refractiveindex.info).

Material	Refractive index (real part)	Refractive index (imaginary part)
Air	1.00	0.00
Aluminum	2.6265	8.3766
Sapphire	1.7606	0.00

3. Two-tint time-domain thermoreflectance setup

The temperature- and width-dependent thermal conductivities are measured using an in house two-tint TDTR setup[4,5], as sketched in Fig. S3. A mode-locked Ti:sapphire laser outputs laser pulses with a wavelength centered around 785 nm and a pulse width of ~ 150 fs at a repetition rate of ~ 80.7 MHz. The laser pulses pass through an optical isolator to prevent any undesired back reflections from destabilizing the laser cavity. After the isolator, the laser pulses are split into a pump arm and a probe arm through the use of a half wave-plate and a polarizing beam splitter (PBS). The pump beam then passes through an electro-optic modulator (EOM) to enable lock-in detection. Two sharp-edged long-pass filters are used on the pump beam path to cut the short wavelength part of the laser spectrum and retain the spectrum with wavelength longer than 791nm. The pump beam then passes through a quarter wave-plate and a linear polarizer and is subsequently focused by a 10x microscope objective onto the sample surface. After the first PBS, the probe beam passes through a mechanical delay stage which regulates the delay time between the pump beam and the probe beam. A sharp-edged short-pass filter is placed on the probe path to cut the long-wavelength part of the laser spectrum and retain the spectrum with wavelength shorter than 781 nm. The probe beam then goes through the quarter wave-plate and the linear polarizer and is focused coaxially with the pump beam onto the sample surface. The combination of the quarter wave-plate and the linear polarizer allows us to control the angle between the laser polarization and the metal grating lines. To create spectrally distinct pump and probe beams, the full-width at half-maximum (FWHM) of the laser source is set to be ~ 12.5 nm. The reflected probe beam from the sample goes through another sharp-edged short-pass filter, which completely rejects the reflected pump beam, before reaching the detector. The lock-in amplifier records the signal as a function of the delay time at the pump modulation frequency. The thermal conductivity of the substrate and the interface conductance

between the metal and the substrate are extracted by matching the experimental signal with the diffusion solution based on the heat equation.

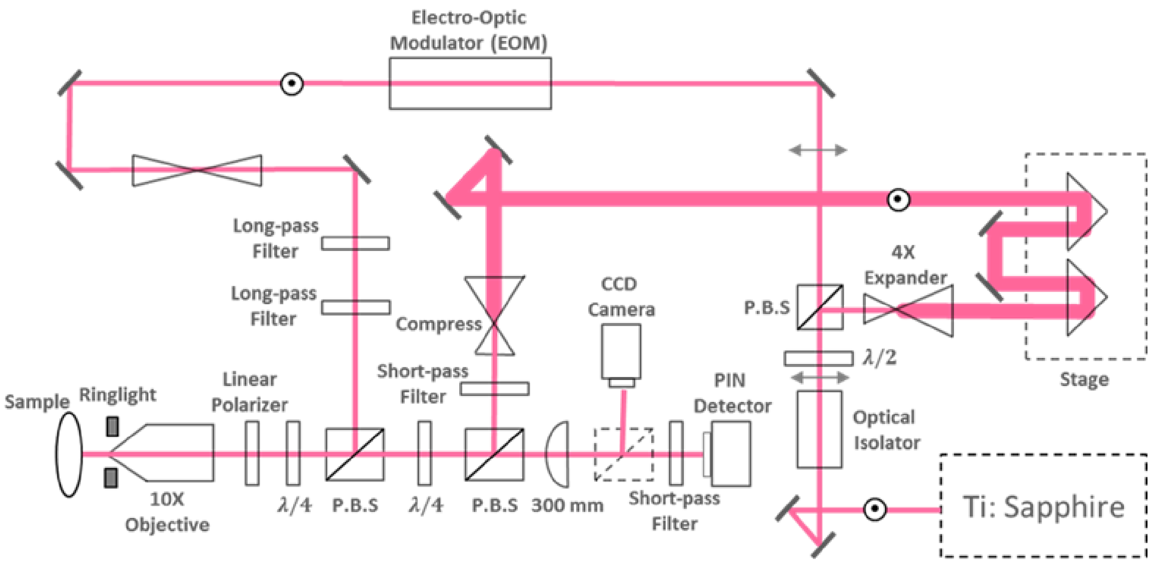

Figure S3. Schematic illustration of our two-tint TDTR setup.

4. Experimental validation of insignificant direct laser transmittance. In addition to the optical simulations discussed before, we experimentally verified the insignificant direct laser transmittance by varying the laser polarization angle relative to the metal grating line direction and measuring how the reflectance signal changed with varying angles. The angle variation leads to variation in the transmitted laser energy into the substrate through the openings between neighboring metal lines. In principle, as the angle increases, a larger portion of the linearly polarized laser energy directly penetrates into the substrate, resulting in an increasing substrate heating effect. Figure S4(a) compares the room-temperature two-tint TDTR signals at different polarization angles obtained from the grating with the smallest (50 nm wide) aluminum line width on a silicon substrate with linearly polarized laser illumination. Zero degree means that the laser polarization is aligned with the metal grating line. The inset shows the early delay-time reflectance signals. Interestingly, the two-tint signals overlap excellently within 30 degree of angle variation and show a clear thermal decay profile. This experimentally confirms that the impact of direct substrate heating is negligible at zero-degree angle illumination.

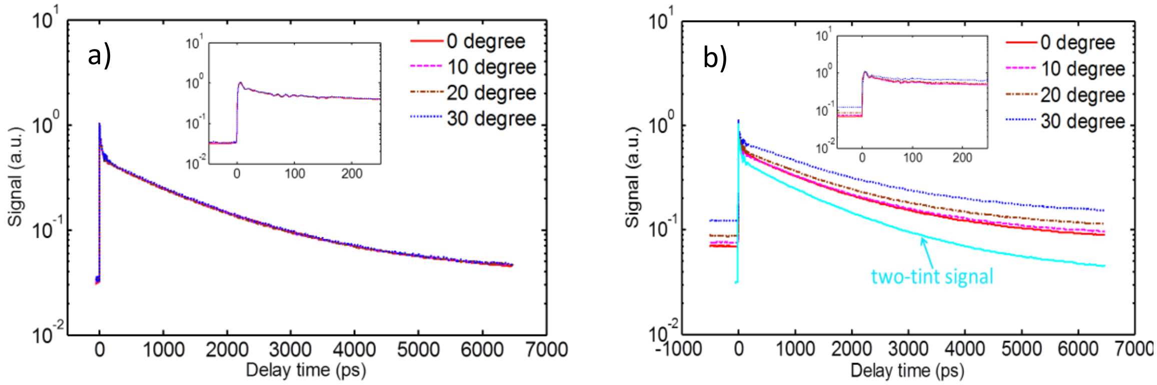

Figure S4. (a) Two-tint TDTR signal as a function of the angle between laser polarization and the metal grating lines for the 50 nm line width grating on silicon substrate, (b) Two-color TDTR signal as a function of the angle between the laser polarization and the metal grating lines for the 50 nm wide grating on silicon substrate.

On the other hand, we performed two-color TDTR[5] (pump wavelength = 400 nm, probe wavelength = 800 nm) measurements with linearly polarized beams on the same 50

nm wide grating sample. As shown in Fig. S4(b), the two-color reflectance signal decays much more slowly than the two-tint signal and rises significantly as the angle increases. This arises because a significant portion of the incident blue laser energy in the two-color TDTR experiments directly enters and heats the substrate and excites electron-hole pairs, resulting in a much slower decay profile due to the smaller temperature difference between the metal grating and the substrate. The heating and excitation become stronger when the angle between the blue laser polarization and the metal grating is increased due to an increased amount of direct laser transmission. The fitted thermal conductivity from two-color TDTR measurements on the 50 nm wide grating sample is approximately 17.0 W/mK at zero degree and decreases constantly as the angle is increased. It is clear that the thermal conductivities derived from the two-color signals, as affected by the direct substrate heating and electron-hole pair generation, are remarkably lower than those derived from two-tint signals (~66 W/mK) and also do not reflect the real quasiballistic thermal transport effects physically induced purely by the finite heater line width since the diffusion heat transfer model used to fit the experimental signal does not account for direct heating and electron-hole pair generation in the substrate. Moreover, the thermal conductivities derived from the two-tint signals have a weak angle dependence within 30 degree of angle variation, again confirming the insignificant direct substrate heating. For heater line width larger than 50 nm, the reflectance signals measured by the two-tint TDTR overlap quite well even for an angle variation larger than 30 degree due to lower laser energy penetration into the substrate relative to heating of the metal lines.

5. Diffusion heat transfer model

In the TDTR measurements, the response from the thermal system is in the linear regime, meaning that a change in the metal surface temperature is linearly related to a change in the metal surface reflectance[5,6]. Measuring the reflectance change at the metal surface is equivalent to measuring the surface temperature since normalized amplitude signals are used. To extract the effective thermal conductivity of the substrate, we match the solution from a diffusion heat transfer model to the experimentally measured reflectance signals[7]. A detailed thermal analysis is given elsewhere[5–7]. Here, we only describe the diffusion model specific to the 1D grating structure. Typically, the sample thermal response is given by a complex transfer function *Z*:

$$Z(\tau) = \sum_{m=-\infty}^{\infty} H(\omega + m\omega_s) e^{jm\omega_s \tau} \quad \text{(S1)}$$

where τ is the delay time between pump and probe beams, ω is the pump beam modulation frequency (~1 MHz – 15 MHz), ω_s is the laser repetition frequency (~80.7 MHz), and $j = \sqrt{-1}$ is the imaginary number. The real part of the transfer function Re(*Z*) and the imaginary part of the transfer function Im(*Z*) correspond to the in-phase and out-of-phase signal component returned by the lock-in amplifier, respectively[5]. The sample frequency response $H(\omega)$ can be obtained by solving the heat equation for the layered system in the frequency domain using a standard transfer matrix method[6]. In particular, since the pump and probe diameters are much larger than the grating line width and period, we assume effectively infinitely large pump and probe spot sizes, thus neglecting the heat conduction along the metal grating direction (x direction as shown in Fig. S5) and using a spatially 1D rectangular heating and probing profile to represent the pump and probe intensity profiles. To account for the discontinuous nature of the metal gratings in the direction perpendicular to the grating (y direction as shown in Fig. S5), we further assume that the in-plane thermal conductivity of the metal transducer is zero, meaning that heat can only diffuse in the cross-plane direction in the metal. Consequently,

when we apply the spatially 1D rectangular heating profile on the metal surface, there is no direct cross-talk between neighboring metal grating lines, mimicking the experimental conditions[8,9]. Using an approach similar to the one described for the 2D square nanodots in Refs. 8 and 9, we obtained the Fourier components for the 1D rectangular wave heating and probing function:

$$X_n = \begin{cases} \frac{\mathrm{w}}{L}, & n = 0 \\ \frac{1}{2\pi n j}\left(1 - e^{-jn\Omega_0 \mathrm{w}}\right), & n \neq 0 \end{cases} \tag{S2}$$

where w is the grating line width, L is the grating period, $\Omega_0 = \frac{2\pi}{L}$ is the fundamental spatial frequency, n is the index for the spatial Fourier transform frequency $k_y = n\Omega_0$.

The sample frequency response is given by:

$$H(\omega) = \sum_n |X_n|^2 (-\frac{D}{C})_n \tag{S3}$$

where C and D are the matrix elements defined in Ref. 8 with the definition of q being that $q = \sqrt{\frac{\sigma_{xy}k_y^2 + C_\mathrm{V} j\omega}{\sigma_z}}$ (σ_{xy}: in-plane thermal conductivity; σ_z: cross-plane thermal conductivity; C_V: volumetric specific heat; k_y: spatial Fourier transform variable in y). Again, the subscript n corresponds to evaluating the arguments at $k_y = n\Omega_0$.

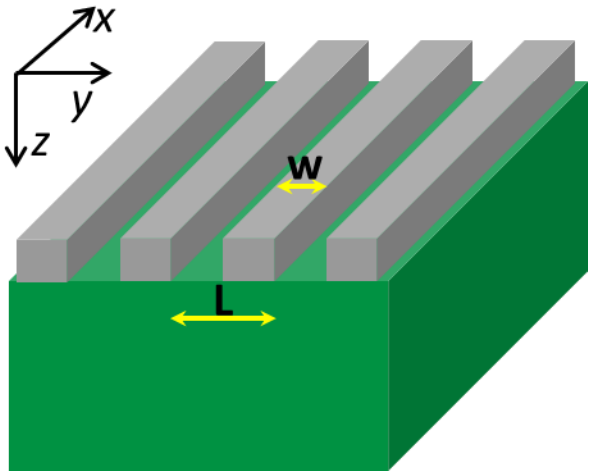

Figure S5. Coordinate system used in the heat transfer model.

6. Measured size-dependent Si thermal conductivities at different temperatures

Table S2 shows the measured size-dependent thermal conductivities of Si at four different temperatures. The measurements were done with the laser beam polarization being parallel to the metal grating nanostructure in order to prevent direct substrate heating effect. We note that for the smallest heater width, the spacing is slightly larger than 150 nm. However, as verified by the angle variation experiments discussed in Section 4 of this Supplementary Information, a spacing slightly larger than 150 nm is still sufficient to prevent significant direct substrate heating. Consistent heater line width and grating period are used in the diffusion heat transfer model to extract the effective thermal conductivity from the measured thermal signal. Also, consistent suppression functions corresponding to the samples' filling fractions are used in the reconstruction process to find the phonon MFP distributions.

Table S2. Measured size-dependent thermal conductivities (W/mK) at different temperatures.

Heater width w (μm)	0.05	0.115	0.22	0.449	0.656	0.87	1.05	2.06	4.057	6.057	8.056	10.058	30	
Grating period L (μm)	0.25	0.30	0.40	0.60	0.80	1.00	1.20	2.20	4.20	6.20	8.20	10.20		k_bulk
T = 200 K	106	179	233	247	251	260	269	269				274	255	274
T = 250 K	86	131	153	170	181	183	188	189				189	190	192
T = 300 K	66.8	99.7	120	135	136	138	139	146	142	146	143	144	143	145
T = 350 K	55.6	81.6	95.9	104	107	108	108	110	112	111	110	112	110	112

7. Measurement sensitivity

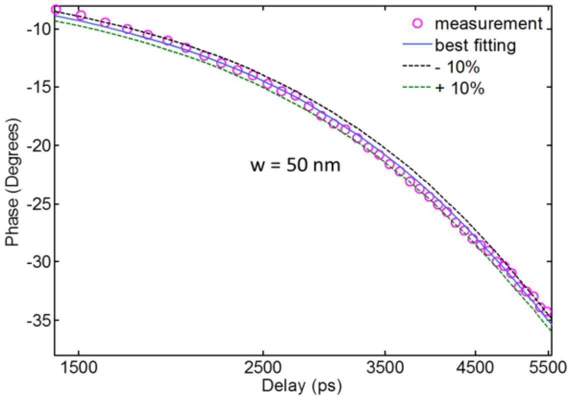

(a)

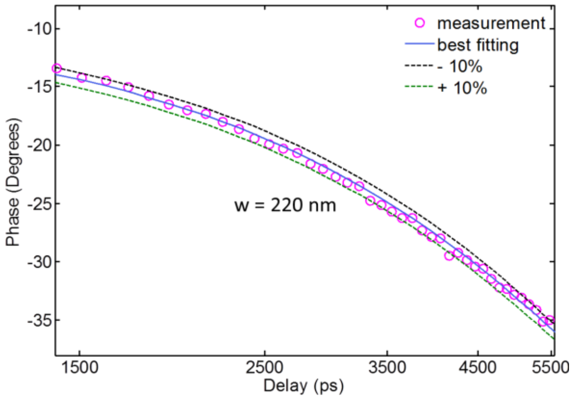

(b)

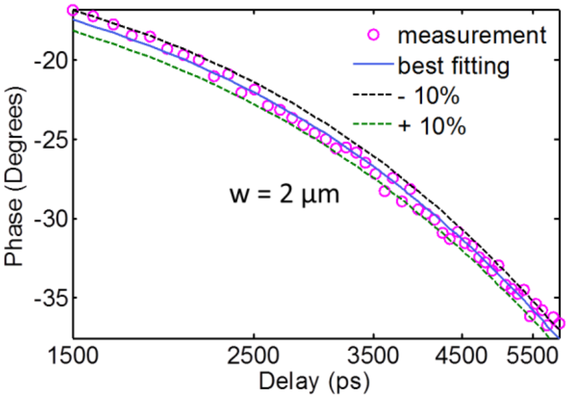

(c)

Figure S7. Measurement data and model predictions for the (a) 50 nm, (b) 220 nm, and (c) 2 μm wide grating samples at room temperature. The circles and the blue solid lines represent the measurement data and the best model fits, respectively. The green and black dashed lines represent the model predictions if the best fitted substrate thermal conductivity is varied by +/- 10%, respectively.

In Fig. S7, we show the measurement sensitivity at room temperature by varying the best fitted substrate thermal conductivity by +/-10%. Similar sensitivities are observed at other examined temperatures in the TDTR experiments.

8. Thermal conductivity accumulation function from DFT calculation

The Si thermal conductivity accumulation function from DFT calculation is obtained by following the same approach described in Ref. 10. However, finer 64 x 64 x 64 k meshes (much denser than the 18 x 18 x 18 k meshes used in Ref. 10) in the reciprocal space are used to do the computation. As shown in Fig. S8, the cumulative MFP distribution data using 64 x 64 x 64 k meshes removes those nonphysical sharp features present in the data computed from an 18 x 18 x 18 k meshes[10]. Our MFP reconstruction result agrees well with the thermal conductivity accumulation function calculated using a denser k mesh.

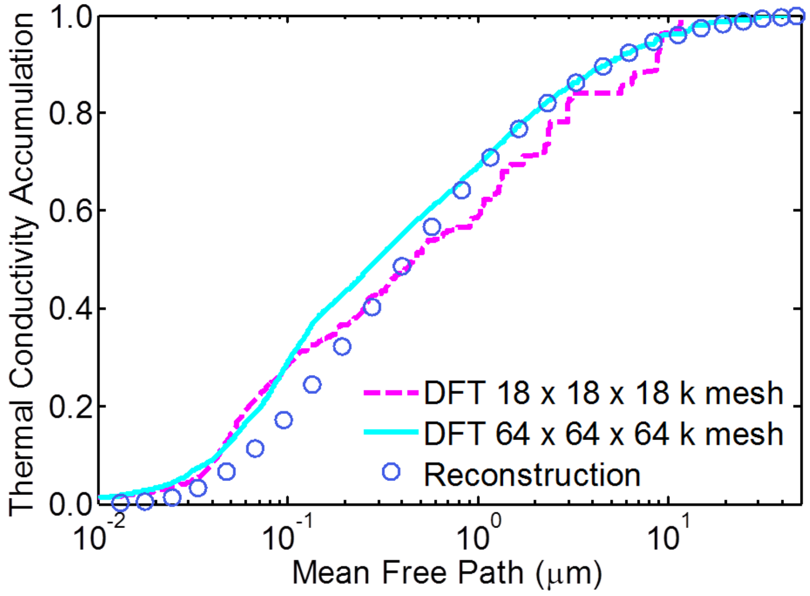

Figure S8. Comparison of Si thermal conductivity accumulation from DFT calculations with different numbers of k meshes and experimental reconstruction at room temperature. The MFP distribution from DFT with an 18 x 18 x 18 k mesh is extracted from Ref. 10. The MFP distribution from DFT with a 64 x 64 x 64 k mesh is computed using the same approach described in Ref. 10.

References

1. COMSOL. *Wave Optics Module Model Library Manual. Version 4.3b* (2013).

2. Hecht, E. *Optics*. (Addison Wesley, 2002).

3. Wang, J. J. *et al.* 30-nm-wide aluminum nanowire grid for ultrahigh contrast and transmittance polarizers made by UV-nanoimprint lithography. *Appl. Phys. Lett.* **89,** 141105 (2006).

4. Kang, K., Koh, Y. K., Chiritescu, C., Zheng, X. & Cahill, D. G. Two-tint pump-probe measurements using a femtosecond laser oscillator and sharp-edged optical filters. *Rev. Sci. Instrum.* **79,** 114901 (2008).

5. Schmidt, A. J., Chen, X. & Chen, G. Pulse accumulation, radial heat conduction, and anisotropic thermal conductivity in pump-probe transient thermoreflectance. *Rev. Sci. Instrum.* **79,** 114902 (2008).

6. Cahill, D. G. Analysis of heat flow in layered structures for time-domain thermoreflectance. *Rev. Sci. Instrum.* **75,** 5119 (2004).

7. Schmidt, A. J. Optical Characterization of Thermal Transport from the Nanoscale to the Macroscale. (2008).

8. Minnich, A. J. Exploring Electron and Phonon Transport at the Nanoscale for Thermoelectric Energy Conversion. (2011).

9. Hu, Y., Zeng, L., Minnich, A. J., Dresselhaus, M. S. & Chen, G. Spectral Mapping of Thermal Conductivity through Nanoscale Ballistic Transport. *Nat. Nanotechnol.* (2015).

10. Esfarjani, K., Chen, G. & Stokes, H. T. Heat transport in silicon from first-principles calculations. *Phys. Rev. B* **84,** 085204 (2011).
